# Emergent and evolving antimicrobial resistance cassettes in community-associated fusidic acid and meticillin-resistant *Staphylococcus aureus*

**DOI:** 10.1016/j.ijantimicag.2015.01.009

**Published:** 2015-05

**Authors:** Matthew J. Ellington, Sandra Reuter, Simon R. Harris, Matthew T.G. Holden, Edward J. Cartwright, Daniel Greaves, Sarah M. Gerver, Russell Hope, Nicholas M. Brown, M. Estee Török, Julian Parkhill, Claudio U. Köser, Sharon J. Peacock

**Affiliations:** a Public Health England, Microbiology Services Division, Addenbrooke's Hospital, Hills Road, Cambridge CB2 0QW, UK; b Wellcome Trust Sanger Institute, Wellcome Trust Genome Campus, Hinxton, Cambridge CB10 1SA, UK; c Department of Medicine, University of Cambridge, Addenbrooke's Hospital, Hills Road, Cambridge CB2 0QW, UK; d Cambridge University Hospitals NHS Foundation Trust, Cambridge CB2 0QQ, UK; e Public Health England, 61 Colindale Avenue, London NW9 5EQ, UK

**Keywords:** CA-MRSA, Antimicrobial resistance, Surveillance, SCC genomics

## Abstract

Fusidic acid is a topical and systemic antimicrobial used for the treatment of staphylococcal infections in hospitals and the community. Sales of fusidic acid and resistance rates among meticillin-resistant *Staphylococcus aureus* (MRSA) doubled between 1990 and 2001. For the following decade, fusidic acid resistance rates among isolates from Addenbrooke's Hospital (Cambridge, UK) were compared with national resistance rates from MRSA bacteraemia surveillance data and with antimicrobial sales data. Sales of fusidic acid remained relatively constant between 2002 and 2012, whilst fusidic acid resistance increased two- and four-fold in MRSA bacteraemias nationally and in MRSA isolates from Cambridge, respectively. A subgroup of MRSA resistant only to fusidic acid increased after 2006 by 5-fold amongst bacteraemias nationally and 17-fold (to 7.7% in 2012) amongst Cambridge MRSA isolates. All of the available local isolates from 2011 to 2012 (*n* = 23) were acquired in the community, were not related epidemiologically and belonged to multilocus sequence typing (MLST) groups ST1, 5, 8, 45 or 149 as revealed from analysis of whole-genome sequence data. All harboured the *fusC* gene on one of six distinct staphylococcal cassette chromosome (SCC) elements, four of which were dual-resistance chimeras that encoded β-lactam and fusidic acid resistance. In summary, fusidic acid-resistant MRSA increased in prevalence during the 2000s with notable rises after 2006. The development of chimeric cassettes that confer dual resistance to β-lactams and fusidic acid demonstrates that the genetics underpinning resistance in community-associated MRSA are evolving.

## Highlights

• Resistance to fusidic acid rose among meticillin-resistant *Staphylococcus aureus* (MRSA) during the 2000s.
• Combined surveillance data and whole-genome sequencing analysis dissected changes in the epidemiology of MRSA.
• Fusidic acid resistance emerged fastest in the community, where it was used the most.
• Resistance occurred in diverse MRSA strains, encoded on staphylococcal cassette chromosome (SCC) elements.
• Compact chimeric dual-resistance cassettes indicate a new mechanism for resistance accrual in MRSA.

## 1 Introduction

The emergence and dissemination of community-associated meticillin-resistant *Staphylococcus aureus* (CA-MRSA) lineages in the community [1] and hospital settings [2] pose a significant clinical problem due to the lack of response to first-line empirical antimicrobials (β-lactams). Alternative agents that can be easily administered in either setting are required, particularly in light of the fact that skin and soft-tissue infections (SSTIs) are now the predominant known cause of MRSA bacteraemia in England (R. Hope, personal communication). One option is fusidic acid, which is licensed in Western Europe, Canada, Australia, New Zealand and numerous countries in Asia (but not in the USA) [3] for the topical treatment of superficial staphylococcal infections affecting the skin and eye as well as for systemic administration for deep-seated infections including those of the bones and joints.

The utility of fusidic acid depends on the rate of resistance, which differs between countries [4,5]. For example, the rate of resistance among *S. aureus* in 2007–2008 in the USA was only 0.3% compared with 7% in Canada and Australia and 11.8% in the UK [4,5]. Fusidic acid inhibits protein synthesis by preventing the turnover of elongation factor G (EF-G) from the ribosome, and resistance usually emerges through point mutation(s) in the chromosomal gene encoding EF-G (*fusA*), which typically confers high-level resistance [6]. More rarely, lower-level resistance can arise via the acquisition of factors that protect the translational machinery (encoded by plasmid-borne genes *fusB* or *fusC*) [5,7].

Fusidic acid resistance among MRSA causing bacteraemia in the UK increased from 1.8% in 1990 to 5.5% in 2001, which accompanied the increased use of this drug from 1.2 tonnes (t) to 3 t annually during the same period [8]. In this study, fusidic acid usage was compared with fusidic acid resistance rates in bacteraemia isolates in the decade from 2002 in England. Fusidic acid resistance in all MRSA (bacteraemia and non-bacteraemia) was investigated by examining data from patients admitted to Addenbrooke's Hospital in Cambridge (UK). This was complemented with a detailed genomic investigation of the resistance mechanisms of MRSA isolates that were resistant to fusidic acid but susceptible to the panel of other drugs commonly tested. Specifically, the hypothesis that fusidic acid resistance had emerged and was disseminating via a mobile genetic element in one or more CA-MRSA lineages was examined.

## 2 Methods

### 2.1 Antimicrobial usage data, and local and national surveillance data

National antimicrobial prescription data collected by IMS Health (London, UK) between 2002 and 2012 were supplied and analysed for changes in the amounts and proportions of the drug that was sold. National surveillance data for MRSA bacteraemias submitted by National Health Service (NHS) trusts to Public Health England via the voluntary reporting system [9] between 2002 and 2013 was accessed. From 2002 to 2013, the overall percentage of MRSA reported with associated fusidic acid susceptibility data was 76% (median annual percentage 80.8%, range 71.8–89.0%), whilst the overall percentage with susceptibility data for fusidic acid, a β-lactam drug, erythromycin, ciprofloxacin, gentamicin and tetracycline was 38.1% (median annual percentage 51.9%, range 23.7–65.2%) (Supplementary Fig. S1). To examine local trends, the microbiology laboratory database at Cambridge University Hospitals NHS Foundation Trust (CUH) was accessed to identify all patients who were MRSA-positive between 2002 and 2013. This demonstrated that >90% of first isolates of MRSA had been tested for fusidic acid plus a β-lactam drug, erythromycin, ciprofloxacin, gentamicin and tetracycline between 2002 and 2013 (Supplementary Fig. S1). Data were collected on the first MRSA-positive sample from each case and included year of isolation, patient location at the time of sampling and sample type. Cases with an MRSA isolate that was resistant to fusidic acid but otherwise susceptible to other routinely tested drugs were identified and were expressed as a proportion of the MRSA tested for fusidic acid per year (see Supplementary Fig. S1).

### 2.2 Local clinical setting and microbiology

CUH is a 1000-bed secondary and tertiary referral hospital. The on-site Clinical Microbiology and Public Health Laboratory provides diagnostic microbiology services to CUH, two additional NHS trusts and three primary care trusts in the area. For CUH, ca. 600 000 clinical specimens are processed per year including screening samples for MRSA, which since 2009 have been taken for all emergency and elective admissions. Antimicrobial susceptibility testing was performed by the diagnostic laboratory at CUH for cefoxitin, erythromycin, ciprofloxacin, gentamicin, tetracycline, rifampicin, fusidic acid and mupirocin using the disk diffusion method as defined by the British Society for Antimicrobial Chemotherapy [10]. Additional testing was performed using a VITEK^®^ 2 instrument (bioMérieux, Marcy-l’Étoile, France) to determine susceptibility to oxacillin, trimethoprim/sulfamethoxazole, linezolid and tigecycline as well as the minimum inhibitory concentration (MIC) to fusidic acid.

### 2.3 Bacterial genome sequencing and sequence analysis

DNA was extracted from *S. aureus*, sequencing libraries were prepared and whole-genome sequencing (WGS) was performed on an Illumina MiSeq instrument (Illumina Inc., San Diego, CA) as previously described [11]. The genome data have been deposited in the European Nucleotide Archive (see Supplementary Table S1). Multilocus sequence typing (MLST) types were assigned from the sequence data [11]. Having established the sequence type (ST), sequence reads were mapped to the relevant reference genome representing four STs (ST1, ST5, ST8 and ST45; accession nos. BX571857, BA000018, CP000255 and BX571856, respectively) using SMALT (https://www.sanger.ac.uk/resources/software/smalt/) [12]. Single nucleotide polymorphisms (SNPs) were identified using a standard approach by removing SNPs with low quality scores and by filtering for SNPs that were present in ≥75% of the mapped reads [11]. Mobile genetic elements were excluded from the resulting whole-genome alignments [13]. Genes encoding antimicrobial resistance were detected by mapping a pseudomolecule that included the known acquired fusidic acid resistance genes in *S. aureus* (Supplementary Table S2) against de novo genome assemblies using SMALT. This allowed the same gene to map multiple times to the assembly using 90% nucleotide identity as the cut-off for detection as described previously [11]. Sequence reads were also mapped against the susceptible variant of the *fusA* gene (encoding the EF-G) to detect mutations conferring resistance towards fusidic acid. Staphylococcal cassette chromosome (SCC) regions were visualised (including sequence coverage and SNP variations) using Artemis [14] and were compared using the Artemis Comparison Tool. The *fusC*-encoding region in the ST8 isolate (MRSA18) was highly fragmented and was not analysed further. Maximum likelihood phylogenies of the SCCs were estimated using RAxML [13].

## 3 Results

### 3.1 Trends in fusidic acid sales and MRSA fusidic acid resistance

**Fig. 1:**
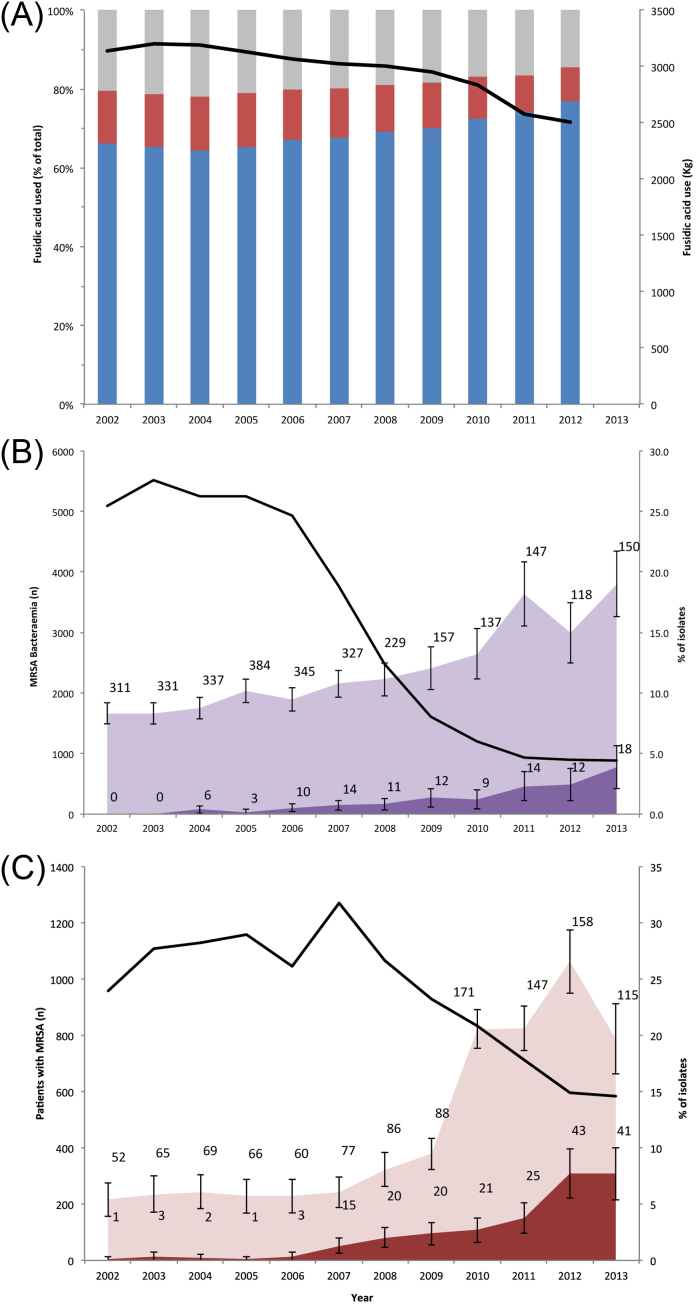
Trends in fusidic acid-resistant meticillin-resistant *Staphylococcus aureus* (MRSA) rates and fusidic acid sales between 2002 and 2013. (A) Percentage of fusidic acid prescribed in the UK for topical use (blue bars) and systemic use (red bars) in the community, and for hospital use (grey bars). These are shown as a proportion of the total drug sold (black line). (B) Number of MRSA bacteraemias in England (black line), the percentage that were resistant to fusidic acid regardless of other antimicrobials (light purple), and the percentage resistant to fusidic acid only (dark purple). (C) The number of MRSA isolated from any body site at Cambridge University Hospitals NHS Foundation Trust (black line), the percentage of the total MRSA that were resistant to fusidic acid regardless of other antimicrobials (pink), and the percentage of MRSA resistant to fusidic acid only (red). The percentages in (B) and (C) were calculated for those isolates for which antimicrobial susceptibility data were available (Supplementary Fig. S1). The bars represent 95% confidence intervals. The number of cases associated with each data point in (B) and (C) are shown.

The amount of fusidic acid prescribed annually in the UK was relatively constant at ca. 3 t between 2002 and 2009, with a decrease to 2.5 t in 2012 (Fig. 1A). The majority (78–85%) of fusidic acid was prescribed in the community, of which between 82% and 90% was for topical use (Fig. 1A). By contrast, topical preparations accounted for 9–13% of fusidic acid sales in the hospital (data not shown). Contrary to the modest decline in overall sales of fusidic acid, the percentage of fusidic acid resistance amongst MRSA bacteraemia isolates in England approximately doubled over the same time period (Fig. 1B). Notably, MRSA bacteraemia isolates that were only resistant to fusidic acid but were otherwise susceptible were not detected in 2002 but steadily increased in number to reach to 2.4% of MRSA bacteraemia isolates by 2012 and 3.9% in 2013 (Fig. 1B). These increases occurred in the context of a six-fold decrease in national MRSA bacteraemias, from a peak of 5522 cases in 2003 to 887 cases in 2013 (Fig. 1B).

To investigate whether these bacteraemia surveillance data were representative for MRSA isolated from other sample sites, the microbiology database at CUH was interrogated. The prevalence of fusidic resistance amongst all MRSA isolated in our centre quadrupled from 5.4% in 2002 (Fig. 1C), supported by a two- to three-fold increase in the number of cases. The proportion of MRSA that were only resistant to fusidic acid increased from 0.2% in 2006 to just over 7% in 2013, supported by a ≥10-fold increase in the number of cases. During the same period, total MRSA cases at CUH (positive from any sample, including carriage) more than halved from a peak of 1270 cases in 2007 to 584 cases in 2013.

### 3.2 Emergence of multiple community-associated MRSA lineages with fusidic acid resistance

To investigate whether the large increase in MRSA that were only resistant to fusidic acid (fusidic acid-mono-resistant MRSA) was due to the expansion of a particular clone, all of the available fusidic acid-mono-resistant MRSA isolates that were isolated and stored at CUH during a period of 14 months starting in November 2011 were sequenced [23 available out of a possible 27, all from different patients (P1–P23)]. Meticillin resistance but susceptibility to other routinely tested drugs is often indicative of CA-MRSA [15]. Examination of the hospital databases revealed that the 23 patients had been admitted to a range of clinical specialities at CUH. The majority (*n* = 15) had no prior visit to hospital within the preceding 12 months, all but 1 case (P8) was MRSA-positive within 48 h of admission, and the median age was low for MRSA carriage (30 years) (Table 1). From this, we concluded that these were sporadic cases and that MRSA colonisation was likely to have originated in the community.

Genetic investigation using WGS revealed that the 23 isolates (designated MRSA1 to MRSA23) from these patients were associated with six different STs from four clonal complexes (CCs) [CC1 (ST1); CC5 [ST5, 149 and 2942 (a single-locus variant of ST149)]; CC45 (ST45); and CC8 (ST8)] (Table 1), demonstrating that multiple lineages of fusidic acid-mono-resistant MRSA had arisen via independent genetic events. Of these STs, five have been reported previously to contain examples of CA-MRSA [16–19]. The relatedness of isolates belonging to the same ST was then investigated by mapping the genomes to a reference genome of the same MLST CC. The two most closely related isolates (MRSA15 and MRSA17, both ST149), isolated 8 weeks apart from paediatric urology and adult oncology patients, respectively, were distinguished by 14 SNPs distributed throughout the core genome (Supplementary Table S3). An estimate based on a similar mutation rate identified in *S. aureus* [20] suggests that these two isolates diverged from a common ancestor over ca. 16 months. These findings are indicative of sporadic MRSA carriage, with no evidence for direct transmission links.

### 3.3 Genetic basis for fusidic acid resistance

Despite being genetically disparate, all of the isolates were classified as having low-level resistance to fusidic acid (MICs of 2–64 mg/L), which is consistent with a transferable resistance mechanism [6,7]. This proved to be the case, since all isolates were positive for *fusC* and had wild-type *fusA*. The genetic regions around *fusC* showed that this gene resided within SCC elements, which are most widely known for harbouring the β-lactam resistance gene *mecA*. Visualisation of the gene content and order within the *fusC*-encoding cassettes revealed six different types of SCC that belonged to two distinct classes [21].

**Fig. 2:**
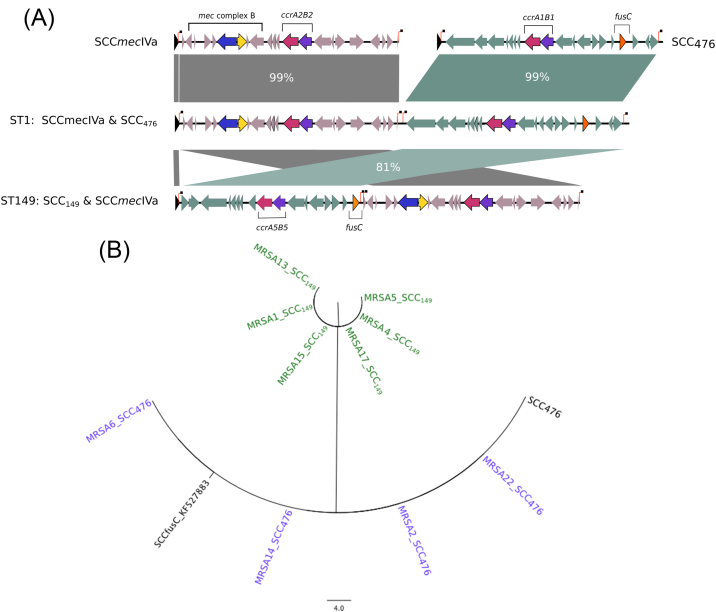
Comparative and phylogenetic analyses of composite islands encoding *mecA* and *fusC*. (A) Staphylococcal cassette chromosome (SCC) elements that encode *fusC* as the only antimicrobial resistance (in teal) were adjacent to a second SCC (SCC*mec*IV, in grey) in composite islands in ST1 and ST149 (and its single-locus variant ST2942). The direct repeat sequences (DR_scc_) that delimit each SCC are shown as vertical red lines topped by a black square. (B) Phylogenetic analysis and comparison with previously identified cassettes SCC_476_ and SCC*fusC* (in black) identifies two distinct SCCs that were unique to ST1 in purple (SCC_476_) or ST149 in green. The novel cassette in found in ST149 isolates was designated SCC_149_.

The first class contained two of the six *fusC*-encoding SCCs, which were adjacent to an SCC*mec*IV [22,23] element forming large composite islands (Fig. 2A). These two composite islands were each unique to a specific lineage (ST1 or ST149/ST2942) (Table 2) and differed in size (45 kb vs. 42 kb, respectively). Within each island, the SCC elements were each flanked by direct repeat sequences (DR_scc_) that demarcated the individual cassettes [23]. A phylogenetic analysis of the 10 isolates with these composite islands indicated that the *fusC* SCC elements were almost identical within a given ST (maximum difference of two SNPs) (Fig. 2B). A comparison was performed between these and previously described composite islands. The cassette in the ST1 isolates (with the exception of MRSA3, see below) was identical to the previously described SCC_476_ (Fig. 2B; Table 2), whereas the cassette that encoded *fusC* in ST149 was novel compared with both of the previously described *fusC*-encoding SCC elements (SCC_476_ and SCC*fusC*) [24,25].

**Fig. 3:**
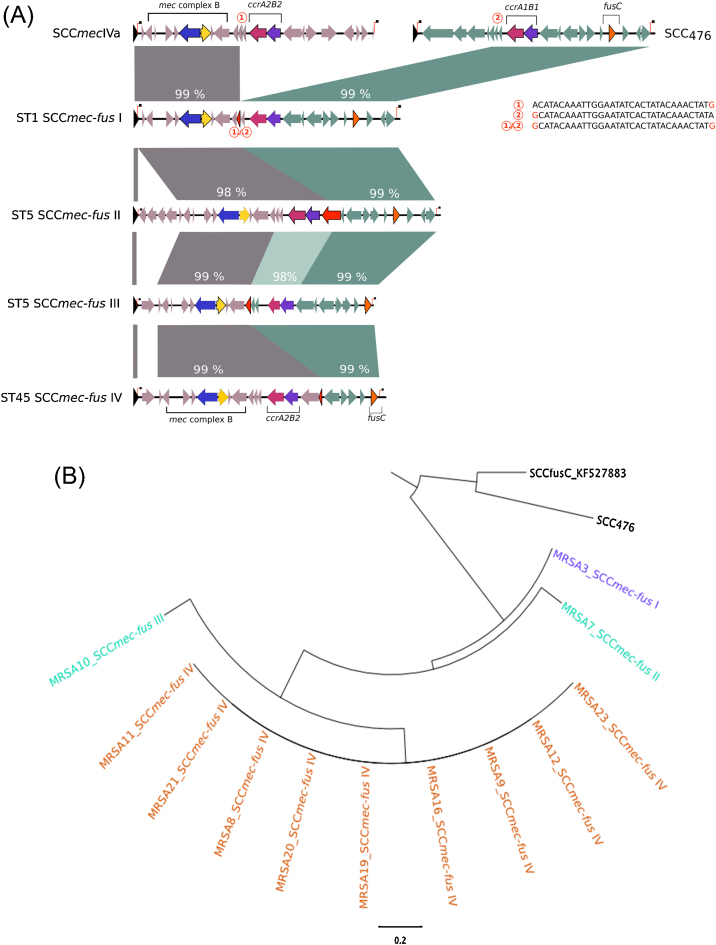
Comparative and phylogenetic analyses of fused staphylococcal cassette chromosome (SCC) elements encoding both *fusC* and *mecA*. (A) SCC elements were formed from portions of SCC*mec* (in grey) and SCC_476_-like cassettes (in teal) and were flanked by direct repeat sequences (DR_scc_). Percentage DNA sequence homologies to the corresponding portion of SCC*mec*IVa or SCC_476_ are shown above each SCC*mec*-*fus*. The change in sequence homology from SCC*mec* to SCC_476_ (denoted and detailed by red numbers in red circles) occurred within coding sequences (shown as red arrow heads). (B) Four different SCCs were identified and were unique to ST1 in purple (SCC_476_), ST5 in cyan and ST45 in orange. The reference cassettes SCC_476_ and SCC*fusC* are shown in black.

The second class contained the four remaining genetic elements, which consisted of a single fused, or chimeric, SCC element that contained both the *mecA* and *fusC* resistance genes, rather than two adjacent SCC elements. These elements were 18–25 kb in size, were flanked by single DR_scc_ sequences at either end of the cassette and were designated SCC*mec*-*fus* I–IV (Fig. 3A). Phylogenetic analysis of these chimeric elements showed that ST45 and ST1 each carried a unique cassette but that isolates within the same ST carried the same element. By contrast, ST5 isolates carried two different cassettes (Fig. 3B; Table 2). The *mecA* or *fusC* portions of the cassettes matched the corresponding parts of SCC*mec*IV and SCC_476_ in the public sequence database (GenBank, 2 September 2014), but we found no cassettes that encoded *mecA* and *fusC* within the same compact and complete SCC region.

In the chimeric element from isolate MRSA3 (SCC*mec*-*fus* I), a 32-bp region that was present in both SCC*mec*IVa and SCC_476_ was identified ((1) and (2) in Fig. 3A), but was in single copy in SCC*mec*-*fus* I. This region was flanked to the left by DNA that was identical to SCC*mec*IVa and to the right by DNA that was identical to SCC_476_. We propose that homologous recombination involving this region led to the junction (“(1)/(2)” in Fig. 3A) and the resultant recombined element SCC*mec*-*fus* I. The other cassettes, SCC*mec-fus* II–IV, showed different homology patterns to the SCC*mec*-like and the SCC_476_ cassettes, indicating independent recombination events that have created new chimeric resistance cassettes in MRSA.

## 4 Discussion

More than 50 years since the first use of fusidic acid, we describe new trends in fusidic acid resistance. MRSA resistant only to fusidic acid increased in prevalence and number between 2002 and 2013. This coincided with the spread of *fusC* on various horizontally transferred chromosomal cassettes, including the newly described SCC*mec*-*fus* chimeric cassettes, in genetically diverse CA-MRSA.

Current data suggest recent global spread of *fusC* MRSA, with an association with the epidemic ST239 clone in Taiwan [25], and the emergence of diverse *fusC* MRSA in New Zealand following increased usage of fusidic acid since 2000 [26]. Previous UK data have shown that between 1990 and 2001 annual sales of this drug more than doubled from 1.2 t to 3 t [8], whilst fusidic acid resistance in MRSA in UK bacteraemia isolates increased from 1.8% to 5.5%. In this study, we examined the decade from 2002, during which the epidemiology of MRSA in the UK changed significantly. Efforts to control hospital-associated MRSA (HA-MRSA) and the subsequent reduction in MRSA bacteraemias since 2007 may have reduced the dissemination of HA-MRSA beyond healthcare settings but has been associated with an expansion of CA-MRSA in the community setting. Fusidic acid sales remained at ca. 3 t until 2009 and subsequently decreased, mostly due to decreased use in hospitals. Nevertheless, fusidic acid resistance amongst MRSA increased to ca. 20%. This is likely to result in more failures of fusidic acid treatment for superficial infections in the community [27,28]. Subsequent empirical systemic treatments (i.e. with β-lactams and prior to culture) will also likely fail and may then result in more severe and disseminated infection. This is of particular relevance given that SSTIs have become the leading source of bacteraemia in England since 2009 (R. Hope, personal communication). This association with SSTIs was consistent with data from CUH, where the majority (71%) of samples taken for clinical reasons (i.e. not including screening samples) were from skin or soft tissue. *fusC* accounted for all of the fusidic acid resistance detected amongst these emergent and genetically diverse MRSA, demonstrating that, regardless of other mechanisms, *fusC* is highly disseminated. *fusC* was found on six different genetic elements, including four elements that were chimeras of two SCCs, one encoding *fusC* and the other encoding β-lactam resistance (*mecA*). These new SCC*mec*-*fus* cassettes were compact dual-resistance cassettes that are defined by normal SCC borders and encoded the machinery necessary for mobilisation. This new mechanism for the accrual of drug resistances into a single compact element has the potential to efficiently transfer multiple resistance genes and could lead to further increases in multidrug resistance amongst *S. aureus*, effectively breathing new life into the evolution of MRSA. This bears a resemblance to the accumulation of drug resistance in Gram-negative bacteria. These genetic rearrangements would have been difficult, if not impossible, to dissect in detail using traditional technologies such as PCR and DNA–DNA hybridisation currently employed for routine molecular epidemiological surveillance. This highlights the importance of introducing routine WGS to complement and enhance existing surveillance schemes.

These findings have two important implications for antibiotic stewardship. First, they reinforce the hazards of using valuable systemic antimicrobials as topical agents widely in the community [29]. The convenience of topical therapy needs to be balanced against the risk of promoting resistance. We believe that it is sensible to restrict the use of topical fusidic acid to short courses and to avoid intermittent use [30] for patients outside hospital, together with close monitoring of local antibiotic susceptibility patterns for evidence of the further spread of fusidic acid-resistant MRSA. Second, traditional approaches to combat antimicrobial resistance, such as decreasing the use of a drug in order to reduce selective pressure, may not be effective based on the current findings. This strategy relies upon different resistance mechanisms being genetically independent of each other. By contrast, genetically linked resistances, such as those we describe, will be co-selected by multiple drugs and any strategies to switch or rotate antimicrobial usage must therefore take account of genetically linked resistances. Most treatment guidelines are based on phenotypic resistance rates alone and consider each antimicrobial class as a discrete unit, further underscoring the value associated with the routine use of WGS for surveillance purposes.

## 5 Conclusions

Fusidic acid resistance amongst MRSA underwent a rapid increase after 2008, more than 50 years after its first use. The heaviest use of fusidic acid occurred in the community, where diverse MRSA that were only resistant to fusidic acid were emerging rapidly, with both known and novel SCCs. The observation of four distinct SCCs that have evolved as chimeras of multiple progenitor cassettes to create compact (<25 kb) dual antimicrobial resistance (SCCs) signals a new mechanism underpinning the development of MRSA and the accrual of multiple resistance genes in *S. aureus*. The cumulative evidence supports the premise that use of fusidic acid in the community is driving the selection of *fusC*-encoding cassettes, including composite SCC*mec-fus* cassettes that can become introduced into hospitals, and highlights the need to control MRSA both in the hospital and community settings.

These findings illustrate the value of combining macro data (i.e. antimicrobial sales and national surveillance data) with local data and targeted molecular investigations that use WGS to dissect changes in MRSA epidemiology. The information we gain with these approaches will help to inform future control strategies for MRSA as it continues to change.

## Funding

This study was supported by grants from the UKCRC Translational Infection Research Initiative and the Medical Research Council [grant no. G1000803] with contributions to the grant from the Biotechnology and Biological Sciences Research Council, the National Institute for Health Research on behalf of the Department of Health, and the Chief Scientist Office of the Scottish Government Health Directorate (to Prof. Peacock); by the Wellcome Trust [grant no. 098051 awarded to the Wellcome Trust Sanger Institute]; by the Health Protection Agency (to Prof. Peacock); and by the NIHR Cambridge Biomedical Research Centre (to Dr Török and Prof. Peacock). EMT is a Clinician Scientist Fellow funded by the Academy of Medical Sciences and the Health Foundation.

## Competing interests

MJE, RH, SMG and NMB are employees of Public Health England. Any views expressed are those of the authors and not necessarily those of Public Health England, the Department of Health or the Wellcome Trust. MJE and CUK have received funding for travel and accommodation from Bruker Daltonics and Janssen, respectively. SJP and JP have received funding for travel and accommodation from Illumina Inc. EMT has received travel and accommodation expenses from Illumina Inc.

## Ethical approval

Ethical approval for this study was received from the Local NHS Research Ethics Committee. Research and Development (R&D) approval for whole-genome sequencing was granted by the R&D Department at Cambridge University Hospitals NHS Foundation Trust.

## Figures and Tables

**Table 1:** Clinical characteristics of patients positive for meticillin-resistant *Staphylococcus aureus* (MRSA) at Addenbrooke's Hospital (Cambridge, UK).

Patient no./isolate no.	Age (years)	Specialty	Reason for presentation	History of skin disease	MRSA isolated within 48 h of admission	Hospital contact in previous 12 months	Isolate sequence type
P1/MRSA1	4	Paediatric ENT	Elective surgery	+	+	−	ST149
P2/MRSA2	22	General surgery	Biliary colic	−	+	−	ST1
P3/MRSA3	20	Obstetrics	Childbirth	−	+	−	ST1
P4/MRSA4	70	Cardiology	Arrhythmia	−	+	−	ST149
P5/MRSA5	36	ENT	Elective surgery	−	+	+	ST2942
P6/MRSA6	83	Orthopaedics	Elective surgery	−	+	−	ST1
P7/MRSA7	44	General medicine	Chest pain	−	+	+	ST5
P8/MRSA8	69	Cardiology	Chest pain	+	−	+	ST45
P9/MRSA9	19	General surgery	Acute pain	−	+	−	ST45
P10/MRSA10	19	A&E	Deep vein thrombosis	+	+	−	ST5
P11/MRSA11	27	ENT	Elective surgery	−	+	−	ST45
P12/MRSA12	49	Plastics	Elective surgery	−	+	−	ST45
P13/MRSA13	21	ENT	Elective surgery	−	+	−	ST149
P14/MRSA14	41	A&E	Trauma	−	+	−	ST1
P15/MRSA15	5	Paediatric urology	Elective surgery	−	+	−	ST149
P16/MRSA16	47	ENT	Elective surgery	−	+	−	ST45
P17/MRSA17	54	Oncology	Day case	−	+	+	ST149
P18/MRSA18	40	Obstetrics	Childbirth	+	+	+	ST8
P19/MRSA19	30	Obstetrics	Childbirth	−	+	−	ST45
P20/MRSA20	2	Paediatric dermatology	Recurrent eczema	+	+	−	ST45
P21/MRSA21	73	Oncology	Day case	+	+	+	ST45
P22/MRSA22	27	Obstetrics	Childbirth	−	+	+	ST1
P23/MRSA23	19	A&E	Trauma	−	+	+	ST45

ENT, ear, nose and throat; A&E, accident and emergency.

**Table 2:** Six genetic elements associated with meticillin and fusidic acid resistance.

Cassette	Sequence type	Patient isolates	Closest published homologues
Composite islands (two adjacent SCCs, encoding either *fusC* or *mecA*)			
SCC_476_ and SCC*mec*IVa	ST1	MRSA2, 6, 14, 22	SCC_476_[24] and SCC*mec*IVa [22]
SCC*mec*IVa and SCC_149_	ST149 + 2942	MRSA1, 4, 5, 13, 15, 17	SCC*mec*IVa [22] and SCC*fusC*[25]
Chimeric cassettes (single cassettes encoding both *fusC* and *mecA*)			
SCC*mec*-*fus* I	ST1	MRSA3	N/A
SCC*mec*-*fus* II	ST5	MRSA7	N/A
SCC*mec*-*fus* III	ST5	MRSA10	N/A
SCC*mec*-*fus* IV	ST45	MRSA8, 9, 11, 12, 16, 19, 20, 21, 23	N/A

SCC, staphylococcal cassette chromosome; N/A, not applicable.
