# Supplementary material for: Emergent and evolving antimicrobial resistance cassettes in community-associated fusidic acid and meticillin-resistant Staphylococcus aureus
Source: Int J Antimicrob Agents. 2015 May;45(5):477–84. doi: 10.1016/j.ijantimicag.2015.01.009 (PMC4415905; doi:10.1016/j.ijantimicag.2015.01.009)
Supplement: Supplementary file 1 [file mmc1.docx]

**Supplementary Table S1**

Accession numbers and study numbers for patients and meticillin-resistant *Staphylococcus aureus* (MRSA) isolates

| **ENA accession #** | **Patient study number** | **Isolate study number** |
| --- | --- | --- |
| ERR108055 | P1 | MRSA1 |
| ERR108056 | P2 | MRSA2 |
| ERR108057 | P3 | MRSA3 |
| ERR108058 | P4 | MRSA4 |
| ERR108059 | P5 | MRSA5 |
| ERR108060 | P6 | MRSA6 |
| ERR108061 | P7 | MRSA7 |
| ERR108062 | P8 | MRSA8 |
| ERR108039 | P9 | MRSA9 |
| ERR108040 | P10 | MRSA10 |
| ERR108041 | P11 | MRSA11 |
| ERR108042 | P12 | MRSA12 |
| ERR108043 | P13 | MRSA13 |
| ERR108044 | P14 | MRSA14 |
| ERR108045 | P15 | MRSA15 |
| ERR108046 | P16 | MRSA16 |
| ERR108047 | P17 | MRSA17 |
| ERR108048 | P18 | MRSA18 |
| ERR108049 | P19 | MRSA19 |
| ERR108050 | P20 | MRSA20 |
| ERR108051 | P21 | MRSA21 |
| ERR108052 | P22 | MRSA22 |
| ERR108053 | P23 | MRSA23 |

ENA, European Nucleotide Archive.
