# Supplementary material for: Emergent and evolving antimicrobial resistance cassettes in community-associated fusidic acid and meticillin-resistant Staphylococcus aureus
Source: Int J Antimicrob Agents. 2015 May;45(5):477–84. doi: 10.1016/j.ijantimicag.2015.01.009 (PMC4415905; doi:10.1016/j.ijantimicag.2015.01.009)
Supplement: Supplementary file 2 [file mmc2.docx]

**Supplementary Table S2**

Sequences used in the resistome pseudomolecule that were mapped with the Illumina data

| **Gene** | **Accession no.** |
| --- | --- |
| *mecA* | X52592 |
| *ermA* | P06699 |
| *ermC* | P13978 |
| *aacA–aphD* | P14507 |
| *aadD* | P05057 |
| *ant1* | P0A0D1 |
| *tetK* | P02983 |
| *dfrG* | C7C2U7 |
| *fusC* | Q6GD50 |
| *ileS-2* | P41972 |
| *fusB* | JF777505 |
| *fusB* | AM292600 |
| *fusA* | DQ866810 |
| *fusA* | **NC003923.1** |
