# Supplementary material for: Emergent and evolving antimicrobial resistance cassettes in community-associated fusidic acid and meticillin-resistant Staphylococcus aureus
Source: Int J Antimicrob Agents. 2015 May;45(5):477–84. doi: 10.1016/j.ijantimicag.2015.01.009 (PMC4415905; doi:10.1016/j.ijantimicag.2015.01.009)
Supplement: Supplementary file 3 [file mmc3.docx]

**Supplementary Table S3**

Whole-genome sequencing (WGS)-derived multilocus sequence typing (MLST) and WGS single nucleotide polymorphism (SNP) typing results for the study isolates

| **Isolate study #** | **ST-appropriate reference** | **#SNPs to ST-appropriate reference** | **Closest within-ST neighbour** | **SNPs to closest neighbour** |
| --- | --- | --- | --- | --- |
| MRSA2 | MW2 | 511 | MRSA14 | 129 |
| MRSA3 | MW2 | 494 | MRSA14 | 61 |
| MRSA6 | MW2 | 496 | MRSA14 | 112 |
| MRSA14 | MW2 | 473 | MRSA22 | 46 |
| MRSA22 | MW2 | 497 | MRSA14 | 46 |
| MRSA7 | N315 | 403 | MRSA10 | 463 |
| MRSA10 | N315 | 373 | MRSA7 | 463 |
| MRSA18 | USA300-FPR3757 | 580 | na | na |
| MRSA8 | MRSA252 | 46468 | MRSA23 | 52 |
| MRSA9 | MRSA252 | 46464 | MRSA12 | 32 |
| MRSA11 | MRSA252 | 46598 | MRSA23 | 37 |
| MRSA12 | MRSA252 | 46503 | MRSA9 | 32 |
| MRSA16 | MRSA252 | 46335 | MRSA23 | 65 |
| MRSA19 | MRSA252 | 46387 | MRSA23 | 65 |
| MRSA20 | MRSA252 | 46324 | MRSA23 | 31 |
| MRSA21 | MRSA252 | 46238 | MRSA8 | 52 |
| MRSA23 | MRSA252 | 46389 | MRSA20 | 31 |
| MRSA1 | N315 | 482 | MRSA17 | 20 |
| MRSA4 | N315 | 479 | MRSA5 | 123 |
| MRSA13 | N315 | 472 | MRSA5 | 73 |
| MRSA15 | N315 | 492 | MRSA17 | 14 |
| MRSA17 | N315 | 483 | MRSA15 | 14 |
| MRSA5 | N315 | 455 | MRSA17 | 73 |

na, not applicable (as no other isolate belonging to ST8 was identified in this work).
