# Supplementary material for: Emergent and evolving antimicrobial resistance cassettes in community-associated fusidic acid and meticillin-resistant Staphylococcus aureus
Source: Int J Antimicrob Agents. 2015 May;45(5):477–84. doi: 10.1016/j.ijantimicag.2015.01.009 (PMC4415905; doi:10.1016/j.ijantimicag.2015.01.009)
Supplement: Supplementary file 4 [file mmc4.docx]

**Supplementary Fig. S1.** Percentages of national meticillin-resistant *Staphylococcus aureus* (MRSA) bacteraemia isolates and MRSA isolated from patients at Cambridge University Hospital (CUH; Addenbrooke’s Hospital) that were tested for fusidic acid susceptibility between 2002 and 2013. National data are shown in purple, with the pale line showing the percentage of MRSA tested for fusidic acid regardless of other antimicrobials and the bold line showing the percentage tested for fusidic acid plus at least five other classes. Data from CUH are shown in red denoting the percentage of MRSA isolates tested for fusidic acid plus at least five other classes of antimicrobials. The bars represent the 95% confidence intervals.
